# The effect of *Withania somnifera* (Ashwagandha) on mental health symptoms in individuals with mental disorders: systematic review and meta-analysis

**DOI:** 10.1192/bjo.2025.10885

**Published:** 2025-10-27

**Authors:** Mattia Marchi, Pietro Grenzi, Antonio Travascio, Daniele Uberti, Edoardo De Micheli, Fabio Quartaroli, Giuseppe Laquatra, Luca Pingani, Silvia Ferrari, Gian M. Galeazzi

**Affiliations:** Department of Biomedical, Metabolic and Neural Sciences, University of Modena and Reggio Emilia, Modena, Italy; Department of Mental Health and Drug Abuse, Azienda USL-IRCCS di Reggio Emilia, Reggio Emilia, Italy

**Keywords:** Anxiety, ashwagandha, depression, mental illness, Withania somnifera

## Abstract

**Background:**

*Withania somnifera* (WS) is considered an adaptogen agent with reported antistress, cognition facilitating and anti-inflammatory properties, which may be beneficial in the treatment of mental disorders.

**Aims:**

This systematic review investigated the efficacy and tolerability of *Withania somnifera* for mental health symptoms in individuals with mental disorders.

**Method:**

The protocol of this review was registered with PROSPERO (CRD42023467959). PubMed, Scopus, PsycINFO, CINAHL, Embase and CENTRAL were searched for randomised controlled trials comparing *Withania somnifera* to any comparator, in people of any age, with any mental disorder. The meta-analyses were based on standardised mean differences (SMDs) and odds ratios with 95% confidence intervals, estimated through frequentist and Bayesian-hierarchical models with random-effects.

**Results:**

Fourteen studies, corresponding to 360 people treated with *Withania somnifera* and 353 controls were included. Anxiety disorders were the predominant diagnostic category. Thirteen trials administered *Withania somnifera* orally (median dose 600 mg/day), one with *Shirodhara* therapy. The median follow-up time was 8 weeks. Although limited by the small number of studies, substantial between-study heterogeneity, and outlier effects, our investigation showed *Withania somnifera* effectiveness in improving anxiety (outlier-corrected SMD: −1.13 (95% CI: −1.65; −0.60), pooled SMD: −1.962 (95% CI: −2.66; −0.57)), depression (SMD: −1.28 (95% CI: −2.40; −0.16) and stress (SMD: −0.95 (95% CI: −1.46; −0.43) symptoms and sleep quality (SMD: −1.35 (95% CI: −1.79; −0.91). The effect size was confirmed using the Bayesian for anxiety but not for depression. No significant difference between *Withania somnifera* and the comparators was found for safety and tolerability.

**Conclusions:**

We found evidence supporting the effectiveness of *Withania somnifera* in treating anxiety symptoms. Future trials should replicate this finding in larger samples and further clarify a possible *Withania somnifera* role in depression and insomnia treatment.

*Withania somnifera* is a perennial herbaceous plant, belonging to the ‘*Solanaceae’* family and native to the Indian areas, which then spread also to Eastern Asia, Southern and Northern Equatorial Africa and Southern-Central Europe.^1^

Although the scientific name of the species is *Withania somnifera*, the plant has been referred to with different names. Probably the most popular name is Ashwagandha which means ’smell of horse’ in Indian dialect (‘aswa’ meaning ‘horse’ and ‘gandha’ meaning ’smell’) in allusion both to the smell of this animal and to the qualities of the herbal extract which can improve strength, vitality and tonicity.^2,3^ The plant is also popularly known in India by different vernacular names like Punir (Hindi), Ashvaganda (Bengal, Bombay), Aksan (Punjab), Amukkira (Tamil) and Tilli (Marathi). *Withania somnifera* is also often referred to as the ‘Indian Ginseng’.^1^ The phytotherapeutic principles of *Withania somnifera* have been discovered and used by Indian and Asian medicine since ancient times; in fact, for thousands of years this plant has been a natural remedy widely used in Ayurvedic and traditional Indian and Asian medicine.^2,4^

Sold as natural supplements, the active constituents can be obtained from the roots, the bark, leaves, seeds and fruits, and used to prepare infusions, tonic and energetics drinks, and in the form of capsules, tablets or drops. The various products based on *Withania somnifera* can be found in herbalists or directly in natural-remedies specialised shops, at variable prices. Alternatively, it is possible to buy *Withania somnifera* supplements by taking advantage of online shopping. Commercial extracts are generally not titled.^5^ The major biochemical constituents of *Withania somnifera* are steroidal alkaloids and lactones, a class of constituents known as withanolides (steroidal lactones with an ergostane skeleton). The withanolides have a structural resemblance with the active constituents of the plant called ‘*Panax ginseng’* and are therefore known as ginsenosides.^1,5^

According to available evidence, the *Withania somnifera* root extract appears to be practically non-toxic. Previous research reported excellent tolerability of *Withania somnifera* after weeks of continuous use, with no significant difference in the incidence of adverse effects compared to a placebo.^6,7^ *Withania somnifera* is classified as an adaptogen; in fact its phytocomplex could modulate the main dopaminergic and serotonergic neurotransmitters, even if further research is needed to verify the specific mechanism of this activity.^8^ *Withania somnifera* preparations are also reported to modulate the γ-amino-butyric acid (GABA) transmission and cholinergic neurotransmission, involved in many central nervous system-related disorders.^8,9^ The active constituents of the plant (withaferin A, sitoindosides VII–X) are reported to have an antioxidant activity which may contribute, at least in part, to the reported antistress, immunomodulatory, cognition facilitating, anti-inflammatory and anti-ageing properties.^1,10^ Given its longstanding use in traditional medicine, it has been used for a wide range of activities for different health conditions. In psychiatry, its supplementation has been tested to treat anxiety, insomnia and stress-induced feelings of depression.^11–14^ A recent review^4^ reported that *Withania somnifera* induced a calming anxiolytic effect that was comparable to lorazepam in three standard anxiety tests, and a previously published, comprehensive, meta-analysis of nutraceutical and phytoceutical compounds in psychiatric disorders suggested *Withania somnifera* for the treatment of anxiety and stress, although with a low level of evidence.^15^ The investigations also support the use of *Withania somnifera* as a mood stabiliser in clinical conditions of anxiety and depression.^4^ Furthermore, the results obtained in a clinical trial and reported in a recent review suggested that the extract of this plant might be effective in combination with standard antidepressants in the treatment of obsessive–compulsive disorder (OCD).^16,17^ In addition, *Withania somnifera* showed promise as a safe and effective treatment for improving immediate and general memory, executive function, attention and information-processing speed in patients with mild cognitive impairment, suggesting possible use in Alzheimer’s disease.^18–20^

Despite the promising properties of *Withania somnifera*, its use in clinical practice has received little attention. Crucially, existing systematic reviews on *Withania somnifera* use in psychiatry have focused on specific disorders and included a limited number of studies. This systematic review aims to bridge this gap by investigating the safety and efficacy of *Withania somnifera* in the treatment of mental health symptoms of people with mental disorders. By providing a comprehensive summary of the existing evidence, our study aims to provide valuable insights to potentially inform future research and advance mental health treatment options.

## Method

This systematic review and meta-analysis was performed according to the Preferred Reporting Items for Systematic Reviews and Meta-Analyses (PRISMA) guidelines.^21^ The protocol of this study was registered with PROSPERO (CRD42023467959).

### Search strategy and selection criteria

We searched the PubMed (Medline), Scopus, PsycINFO, CINAHL, Embase and CENTRAL (covering also the International Clinical Trials Registry Platform (ICTRP) and clinicaltrials.gov) databases until 15 May 2025, using the strategy outlined in Supplemental Table 1 available at https://doi.org/10.1192/bjo.2025.10885. No restrictions regarding language of publication or publication date were set. All randomised controlled trials (RCTs) comparing *Withania somnifera* used as a monotherapy or as an add-on treatment to placebo or other active comparators, in people of any age, with any mental disorder were eligible for the review. Diagnosis was defined according to standard operational diagnostic criteria according to the DSM^22^ or ICD^23^ or validated psychometric tools. Studies were excluded if the Population, Intervention, Comparison, Outcomes and Study framework did not fit with that defined in the review protocol in PROSPERO (CRD42023467959). Specifically, studies that considered a sample of healthy volunteers were excluded. Similarly, participants with exclusively physical conditions, dementia or substance use disorder were excluded, unless they had a co-occurring mental disorder. Studies where all participants received at least one dose of *Withania somnifera*, or that did not provide post-treatment assessment of psychopathology were also excluded. No other limits on participants’ characteristics, concurrent treatment or comorbidity were set. We excluded qualitative studies, case reports, case series and reviews, although the reference lists of the reviews have been screened to identify any potentially eligible studies that could have been missed during the literature search. We only included studies published in peer-reviewed journals, excluding conference abstracts and dissertations. If data from the same trial were published in multiple papers, we considered only the publication reporting more complete information or, in case of parity in this criterion, the largest sample size, to maximise the power of the analyses. Sample overlap was ruled out through a careful check of the trial registration codes as well as the place and year(s) of sampling.

### Data collection and extraction

Two review authors (M.M. and P.G.), working independently, screened all retrieved articles in the original search for inclusion, first on the title, followed by the abstract and then analysed full texts to check compliance with inclusion/exclusion criteria. All disagreements were explored until consensus was reached, and if consensus was not possible, another member of the team was consulted (G.M.G.).

Data extraction started on 12 December 2023. For each eligible trial, the two review authors independently extracted the following information: (a) study characteristics (first author last name, year of publication, country, study setting, number of participants randomised in each arm, number of participants with outcome assessment and duration of the trial); (b) participant characteristics (age, gender, psychiatric diagnoses and stage of illness, symptoms severity at baseline and on-going psychiatric treatment); (c) intervention details (comparator used, prescribed *Withania somnifera* dosage and bioactives’ concentration in the *Withania somnifera* extracts, frequency of administration, route of administration and co-interventions) and (d) outcome measures of interest and time of data collection. Extraction sheets for each study were cross-checked for consistency and any disagreement was resolved by discussion within the research group.

### Outcome measures

For our primary analyses we considered efficacy, which we measured as the difference in the post-treatment score of mental health symptoms among treatment and control arms. Finally, we considered tolerability as: the rate of dropouts due to any cause, the rate of dropouts due to serious adverse effects, the overall rate of side effects and the rate of death, in each trial arm. We assessed those outcomes available at the times closest to the median follow-up time across the studies.

### Statistical analyses

Where possible, we summarised quantitative data among studies using meta-analyses. We used inverse-variance models with random effects to summarise both continuous and dichotomous outcome data.^24^ For continuous outcome data, we calculated the Hedges’ *g* standardised mean differences (SMDs) and the corresponding 95% confidence intervals; for dichotomous outcome data, we calculated the pooled odds ratios and the corresponding 95% CIs.^25^ We used data from the intention-to-treat analyses for both continuous and dichotomous outcomes. The results were summarised using forest plots. Standard Q tests and the *I* ^2^ statistic (i.e. the percentage of variability in prevalence estimates attributable to heterogeneity rather than sampling error or chance, with values of *I* ^2^ ≥75% indicating high heterogeneity) were used to assess between-study heterogeneity.^26^

When the meta-analysis included at least ten studies,^27^ we performed funnel plot analysis and the Egger test to test for publication bias. If analyses showed a significant risk of publication bias, we would use the trim-and-fill method to estimate the number of missing studies and the adjusted effect size.^28–31^ For meta-analysis including at least ten studies, univariable meta-regression analysis was performed to examine sources of between-study heterogeneity on a range of study pre-specified characteristics (i.e. participants’ gender, age in the entire sample; country where the study was conducted; trial duration; *Withania somnifera* dose; concentration of withanolides (i.e. the main active component of *Withania somnifera* extracts) and concurrent medication). Sensitivity analyses included leave-one-out and subgroup meta-analyses using diagnosis as the grouping variable.

When high between-study heterogeneity persisted despite sensitivity analyses, we conducted Bayesian hierarchical meta-analyses to estimate the SMD with 95% credible intervals (95% CrI) using weakly informative priors. This analytic extension was not pre-specified in the PROSPERO-registered protocol and was introduced post hoc in response to peer-review suggestions, to further assess the robustness of the findings given the extent of between-study heterogeneity observed in some comparisons. Specifically, we applied a normal(0,1) prior for the intercept and a cauchy(0,0.5) prior for the s.d. of the random effects.^32^ As a sensitivity analysis, we also tested an alternative model using an exponential(1) prior for the s.d. to evaluate the robustness of posterior estimates to prior specification.^33^ Overall, with this approach we aimed to assess the robustness of the heterogeneity estimates (τ) and the overall pooled effect under different plausible assumptions about the distribution of the true effect sizes across studies.

The analyses were performed using *meta*, *metafor* and *brms* packages in R (version 4.4.0, for MacOS).^34–37^ Statistical tests were two-sided and used a significance threshold of *p*-value <0.05.

### Risk of bias assessment and the GRADE

Bias risk in the included studies was independently assessed by two reviewers (M.M. and F.Q.), using the Cochrane risk of bias tool.^38^ All disagreements were discussed until consensus, and if necessary, another member of the team was consulted (G.M.G.). Each item on the risk of bias assessment was scored as high, low or unclear, and the GRADE tool was used to assess the overall certainty of evidence.^39^ Further information is available in the Supplementary Material.

### Deviations to the PROSPERO protocol

The main deviation from the PROSPERO-registered protocol was the inclusion of Bayesian hierarchical meta-analyses, which were introduced post hoc in response to peer-review feedback as detailed in the statistical analyses section.

## Results

### Study characteristics

**Fig. 1:**
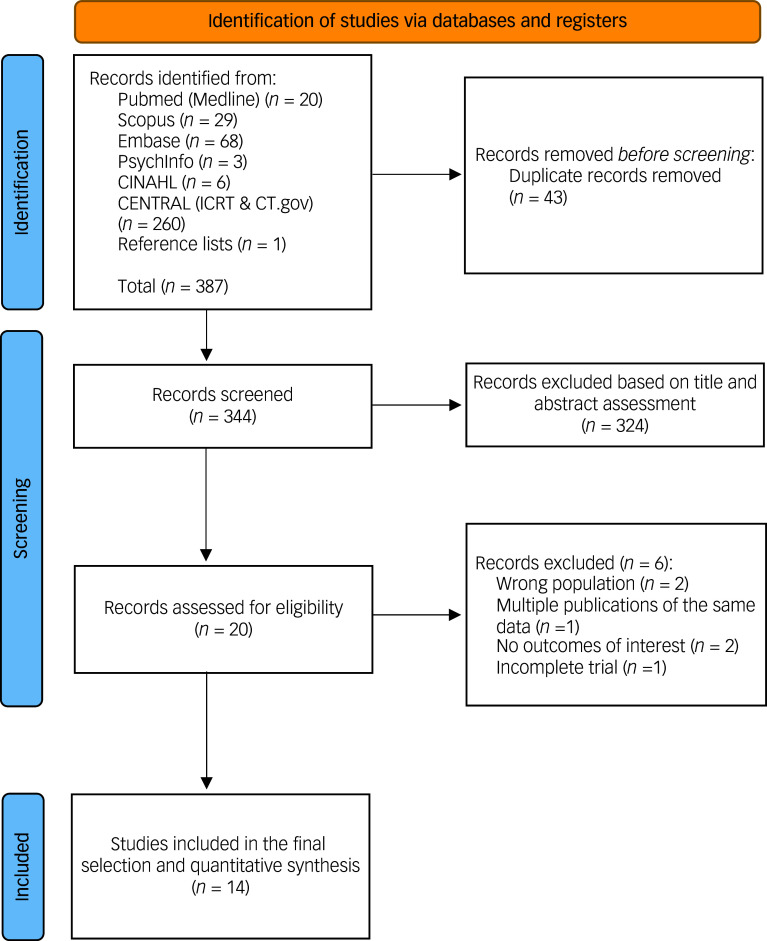
Preferred Reporting Items for Systematic Reviews and Meta-analyses flow diagram.

As shown in Fig. 1, from 387 records screened on title and abstract, 20 full texts were analysed. One additional RCT was found on clinicaltrials.gov but this was an incomplete trial due to inability to reach the target sample size due to COVID-19,^40^ therefore this was not included in our final selection. The review process led to the selection of 14 studies^17,41–53^ referring to 14 independent samples, corresponding to a total of 713 people (of which 360 treated with *Withania somnifera* and 353 controls), which were included in the quantitative synthesis (see also Supplementary Table 2 reporting the list of studies excluded after full-text review, along with the reason for exclusion).

The trials were conducted in 4 countries: India (*n* = 8; 57.2%), Iran (*n* = 3; 21.4%), USA (*n* = 2; 14.3%) and Canada (*n* = 1; 7.1%). The overall percentage of females across the studies was 45.7%, mean age was 38.3 (s.d.: 10.9) years, with only one study (7.1%) involving underage participants. The most common diagnostic category was anxiety disorders (*n* = 5; 35.7%), followed by insomnia and chronic stress (each *n* = 2; 14.3%), major depressive disorder (MDD), bipolar disorder, schizophrenia and schizo-affective disorder, attention-deficit hyperactivity disorder (ADHD) and OCD, each *n* = 1 (7.1%). The clinical severity at the baseline was symptomatic or mild/moderate for most of the studies, except for one study performed on participants with moderate/high severity anxiety and one on participants with bipolar disorder in clinical remission. In 13 trials (92.9%) *Withania somnifera* was administered orally with daily dosing ranging from 10 to 12 000 mg (median 600 mg). One trial (7.1%) administered *Withania somnifera* with Shirodhara (oil dripping) therapy. Withanolides minimum concentration in the *Withania somnifera* extracts ranged from 1.5 to 8%, although this information was not reported by six studies. *Withania somnifera* was administered as add-on treatment in seven trials (50.0%), as the sole therapeutic agent in four trials (28.6%) and three trials (21.4%) did not report information on concurrent medication. For the studies administering *Withania somnifera* as add-on treatment, the concurrent treatment consisted of selective serotonin reuptake inhibitor (SSRI) antidepressants in two studies, mood stabilisers, antipsychotics, anti-ADHD medications without other specification and phytotherapeutic regimen with *Nardostachys jatamansi* and *Lavandula stoechas*, each *n* = 1. Most of the trials used placebo as the comparator (*n* = 12; 85.7%), one study (7.1%) used a combination of placebo and cognitive behavioural therapy (CBT) and one study (7.1%) considered the ongoing phytotherapeutic regimen alone as the control group. The median trial’s duration was 8 weeks, ranging from 6 to 12 weeks. The outcomes reported by the trials were: anxiety (*n* = 10), depression (*n* = 5), sleep (*n* = 4), stress (*n* = 3) and mania, psychotic symptoms and OCD symptoms (each *n* = 1). The main characteristics of the studies included in the review are summarised in Table 1 (see also Supplementary Table 3 for information on the composition of *Withania somnifera* extracts used in each study and Supplementary Table 4 for details on the outcome measures used in each study).

**Table 1:** Characteristics of the included studies

Author, year (trial ID)	Study design	Country	Setting	Diagnosis *(clinical severity)*	Duration	*N* T/*N* C	% Female	Mean age (s.d.) or range	T	C	Concurrent treatment	Outcomes reported
Andrade et al, 2000^41^ (NR)	RCT	India	Out-patient clinic	Anxiety *(Symptomatic)*	6 weeks	20/19	38.5	41.3 (13.8)	WS 500 mg po daily	PBO (NR)	Any stable treatment	Anxiety
Chengappa et al, 2013^42^ (NCT00761761)	RCT	USA	Out-patient clinic	Bipolar disorder I, II, or NOS *(Remission)*	8 weeks	24/29	61.7	46.4 (10.3)	WS 500 mg po daily	PBO (inert filler)	Mood stabiliser	Depression, mania or anxiety
Chengappa et al, 2018^43^ (NCT01793935)	RCT	USA	Out-patient clinic	SZ, schizo-affective disorder *(Symptomatic)*	12 weeks	33/33	48.5	46.3 (12.1)	WS 1000 mg po daily	PBO (inert filler)	AP	Depression, psychotic symptoms
Choudhary et al, 2017^44^ (NR)	RCT	India	Out-patient clinic	Chronic stress *(Symptomatic)*	8 weeks	25/25	26.9	18-60	WS 600 mg po daily	PBO (inert filler)	None	Stress
Cooley et al, 2009^45^ (ISRCTN78958974)	RCT	Canada	Out-patient clinic	Anxiety *(Moderate to high severity)*	12 weeks	36/39	63	51.7 (9.6)	WS 600 mg po daily + adult multi-vitamin	CBT + PBO (inert filler)	None	Anxiety
Fuladi et al, 2021^46^ (IRCT20180615040105N1)	RCT	Iran	NR	GAD *(NR)*	6 weeks	18/22	45	40.2 (8.8)	WS 1000 mg po daily	PBO (lactose)	SSRI	Anxiety
Fulzele et al, 2014^47^ (NR)	RCT	India	NR	MDD *(Mild to moderate severity)*	6 weeks	15/15	NR	20-65	WS, Shirodhara oil (dose NR)	NA	*Nardostachys jatamansi* and *Lavandula stoechas*	Depression
Hosseini et al, 2019^48^ (IRCT201506215280N18)	RCT	Iran	Out-patient clinic	ADHD *(NR)*	6 weeks	14/14	39.3	9.5 (1.6)	WS 10 mg po daily	PBO (NR)	Anti-ADHD treatment	Anxiety
Jahanbakhsh et al, 2016^17^ (IRCT2015070523079N1)	RCT	Iran	NR	OCD *(NR)*	6 weeks	15/15	90	33.1 (10.8)	WS 1000 mg po daily	PBO (lactose)	SSRI	OCD symptoms
Khyati et al, 2013^49^ (NR)	RCT	India	NR	GAD *(NR)*	8 weeks	44/42	NR	16-60	WS 12000 mg po daily	PBO (wheat flour)	NR	Anxiety
Langade et al, 2019^50^ (NR)	RCT	India	Out-patient clinic	Insomnia *(Symptomatic)*	10 weeks	40/20	22.5	39.2 (5.4)	WS 600 mg po daily	PBO (starch)	NR	Anxiety, sleep quality
Langade et al, 2021^51^ (CTRI/2019/03/018074)	RCT	India	Out-patient clinic	Insomnia *(Symptomatic)*	8 weeks	20/20	NR	37.3 (6.4)	WS 600 mg po daily	PBO (starch)	None	Anxiety, insomnia
Majeed et al, 2023^52^ (CTRI/2022/05/042640)	RCT	India	NR	Depression or Anxiety *(Mild to moderate severity)*	12 weeks	34/36	38.6	40.7 (11.3)	WS 500 mg po daily + 5 mg 95% piperine (*Piper nigrum*)	PBO (cellulose)	NR	Depression, anxiety or sleep quality
Pandit et al, 2024^53^ (CTRI/2019/11/022100)	RCT	India	Out-patient clinic	Chronic stress *(Symptomatic)*	8 weeks	22/24	28.6	35.1 (10.3)	WS 500 mg po daily	PBO (NR)	None	Depression, anxiety, sleep quality or stress

ADHD, attention-deficit hyperactivity disorder; AP, antipsychotics; C, control; CBT, cognitive–behavioural therapy; GAD, generalised anxiety disorder; MDD, major depressive disorder; mg, milligram; *N*, number; NA, not applicable; NOS, not otherwise specified; NR, information not reported; OCD, obsessive–compulsive disorder; PBO, placebo; po, per os (by mouth/orally); RCT, randomised controlled trial; SSRI, selective serotonin reuptake inhibitor; SZ, schizophrenia; T, treatment; WS, *Withania somnifera*.

### Effect of *Withania somnifera* on anxiety symptoms

**Fig. 2:**
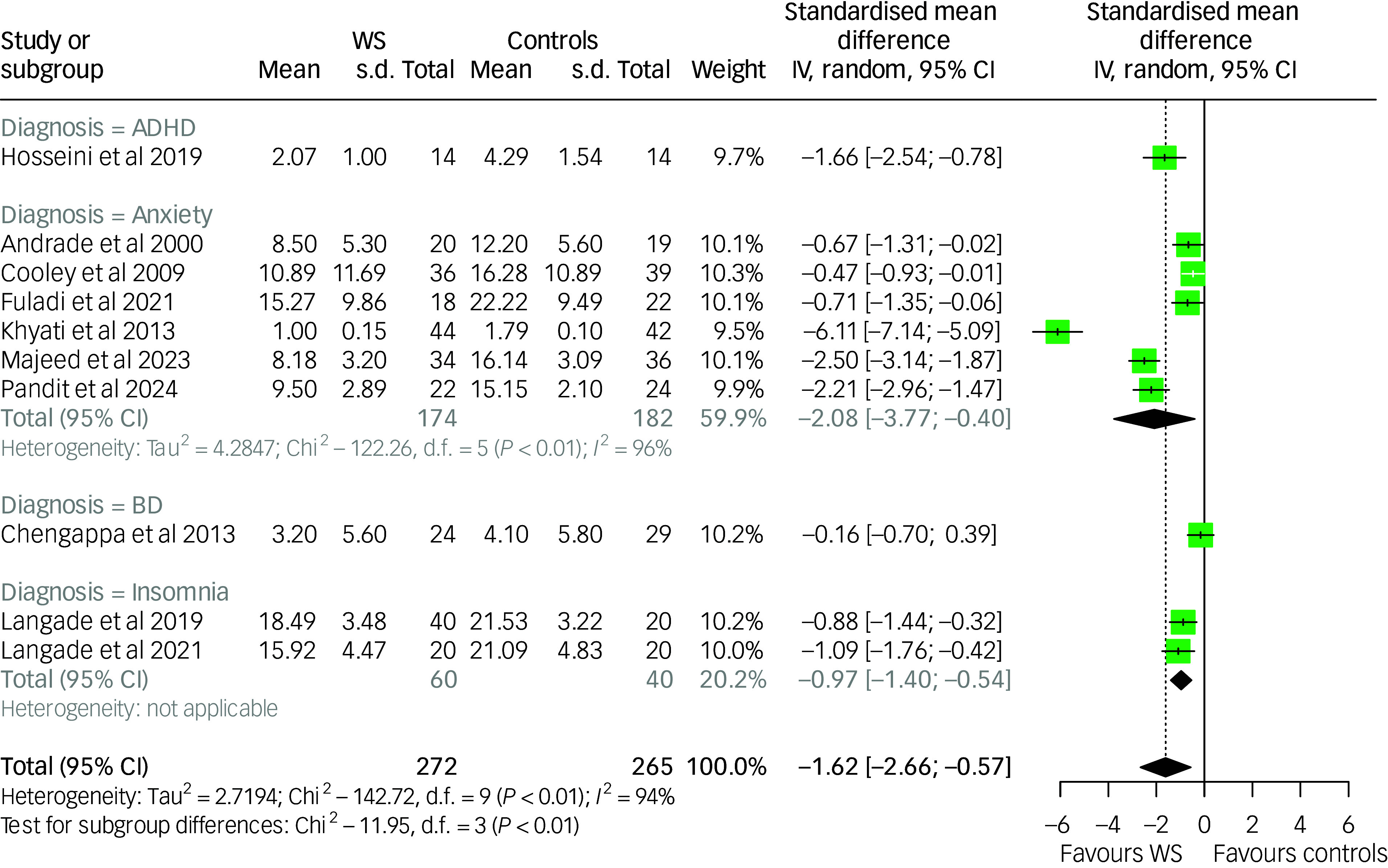
Forest plot of anxiety among *Withania somnifera* and control groups: overall and by diagnostic subgroup. ADHD, attention-deficit and hyperactivity disorder; BD, bipolar disorder; IV, inverse variance; WS, *Withania somnifera*. The measures of anxiety across the studies were scaled as lower scores indicate lower anxiety symptoms. Reference to the included studies (in alphabetical order): Andrade et al, 2000^41^; Chengappa et al, 2013^42^; Cooley et al, 2009^45^; Fuladi et al, 2021^46^; Hosseini et al, 2019^48^; Khyati et al, 2013^49^; Langade et al, 2019^50^; Langade et al 2021^51^; Majeed et al, 2023^52^; Pandit et al, 2024.^53^

The meta-analysis of the effect of *Withania somnifera* on anxiety symptoms included ten studies and yielded significant evidence supporting the efficacy of the treatment (SMD: −1.62 (95% CI: −2.66; −0.57)), although with evidence of high between-study heterogeneity (*I* ^2^: 94%; *p*-value <0.001). Subgroup meta-analysis by diagnostic category revealed a significant between-group difference (*p*-value: 0.008); however, the pooled estimates indicated treatment effectiveness across most conditions, except for the single study conducted in individuals with bipolar disorder, which did not show a significant effect. The results are summarised in Fig. 2.

Our investigation found evidence of publication bias. Notably, most studies reported outcomes favouring *Withania somnifera*, one showed a null effect and none reported negative results. This can be seen in the funnel plot displayed in Supplementary Figure 1 and was confirmed by Egger’s test *p*-value <0.001. However, the estimate of the effect size obtained by inputting missing studies into the funnel plot until symmetry is reached using Duval and Tweedie’s trim-and-fill procedure was highly similar (SMD: −1.62 (95% CI: −2.67; −0.57)).

To investigate the source of heterogeneity, leave-one-out analysis and meta-regression were performed.

Leave-one-out analysis, in which the meta-analysis was serially repeated after the exclusion of each study, found that by excluding the study from Khyati et al^49^ the effect size decreased more than 25%, remaining statistically significant (SMD: −1.13 (95% CI: −1.65; −0.60); *I* ^2^: 85%). Interestingly, this study used the highest dose of *Withania somnifera*, which was more than ten times higher than the other trials. Instead, excluding other studies produced overall inconsequential changes (≤15%) in both the pooled estimate and heterogeneity. The results of leave-one-out analysis are available in Supplementary Table 5.

Meta-regression analyses were performed on the following variables, potentially associated with heterogeneity: (a) the mean age of participants; (b) the percentage of females in the total sample; (c) the country where the study was conducted; (d) *Withania somnifera* dose administered; (e) an interaction term of *Withania somnifera* dose × withanolides % in the extract used; (f) trial duration and (g) *Withania somnifera* use as monotherapy or add-on treatment. In the univariable meta-regression model, the variables resulting significantly associated with the estimate of the treatment effect was the *Withania somnifera* dose, with a larger anxiolytic effect observed in trials administering *Withania somnifera* at a higher dose (unstandardised regression coefficient (B: −0.004 (95% CI: −0.007; −0.001)) and the use as add-on treatment (B: −0.753 (95% CI: −1.47; −0.038)). It should be noted that this result might be strongly influenced by the outlier effect played by the study from Khyati et al^49^ which used a dose of *Withania somnifera* more than ten times higher than the median dose. Univariable meta-regression results are displayed in Supplementary Table 6.

**Fig. 3:**
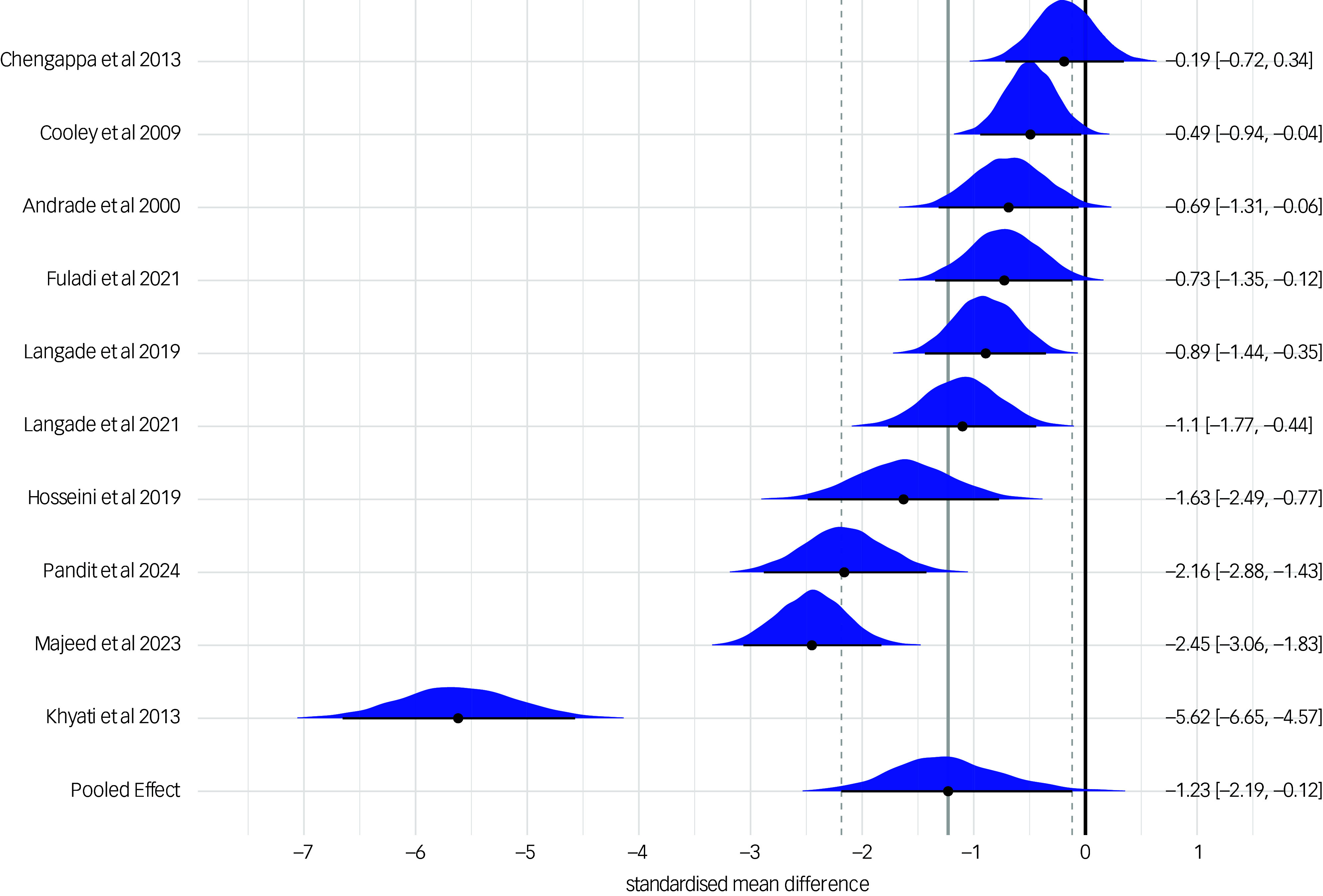
Bayesian forest plot of study-level and pooled posterior estimates of *Withania somnifera* efficacy on anxiety. The measures of anxiety across the studies were scaled as lower scores indicate lower anxiety symptoms. Reference to the included studies (in alphabetical order): Andrade et al, 2000^41^; Chengappa et al, 2013^42^; Cooley et al, 2009^45^; Fuladi et al, 2021^46^; Hosseini et al, 2019^48^; Khyati et al, 2013^49^; Langade et al, 2019^50^; Langade et al 2021^51^; Majeed et al, 2023^52^; Pandit et al, 2024.^53^

The effect of *Withania somnifera* on anxiety symptoms was further examined using a Bayesian hierarchical meta-analysis, which confirmed a statistically significant effect of the treatment (SMD: −1.23 (95% CrI: −2.19; −0.12)). The estimated between-study heterogeneity (τ) was 1.71 (95% CrI: 1.04; 2.82). This model applied weakly informative priors: a normal(0,1) prior for the intercept and a cauchy(0,0.5) prior for the standard deviation of the random effects and converged at 4000 iterations. Results are shown in Fig. 3. For sensitivity analysis, the model was re-estimated using an alternative prior for τ (i.e. exponential(1)). Results were consistent across priors, with stable posterior distributions for both τ and the overall treatment effect (see Supplementary Figure 2). Model comparison using leave-one-out cross-validation indicated similar predictive performance (difference in Expected Log Pointwise Predictive Density (∆ELPD): −0.1). Given the conservative nature of the cauchy(0,0.5) prior, we retained it in the final model.

Finally, we included a *Withania somnifera* dose as a moderator in the Bayesian model. To enhance interpretability, we log-transformed the dose variable and centred the intercept at the median dose of 600 mg/day. At this reference dose, the model estimated a significant treatment effect on anxiety symptoms (SMD: −1.27 (95% CrI: −2.17; −0.21)). However, we found no evidence of a statistically significant increase in treatment efficacy for doses above the median (B: −0.50 (95% CrI: −1.19; 0.16)).

### Effect of *Withania somnifera* on depression symptoms

**Fig. 4:**
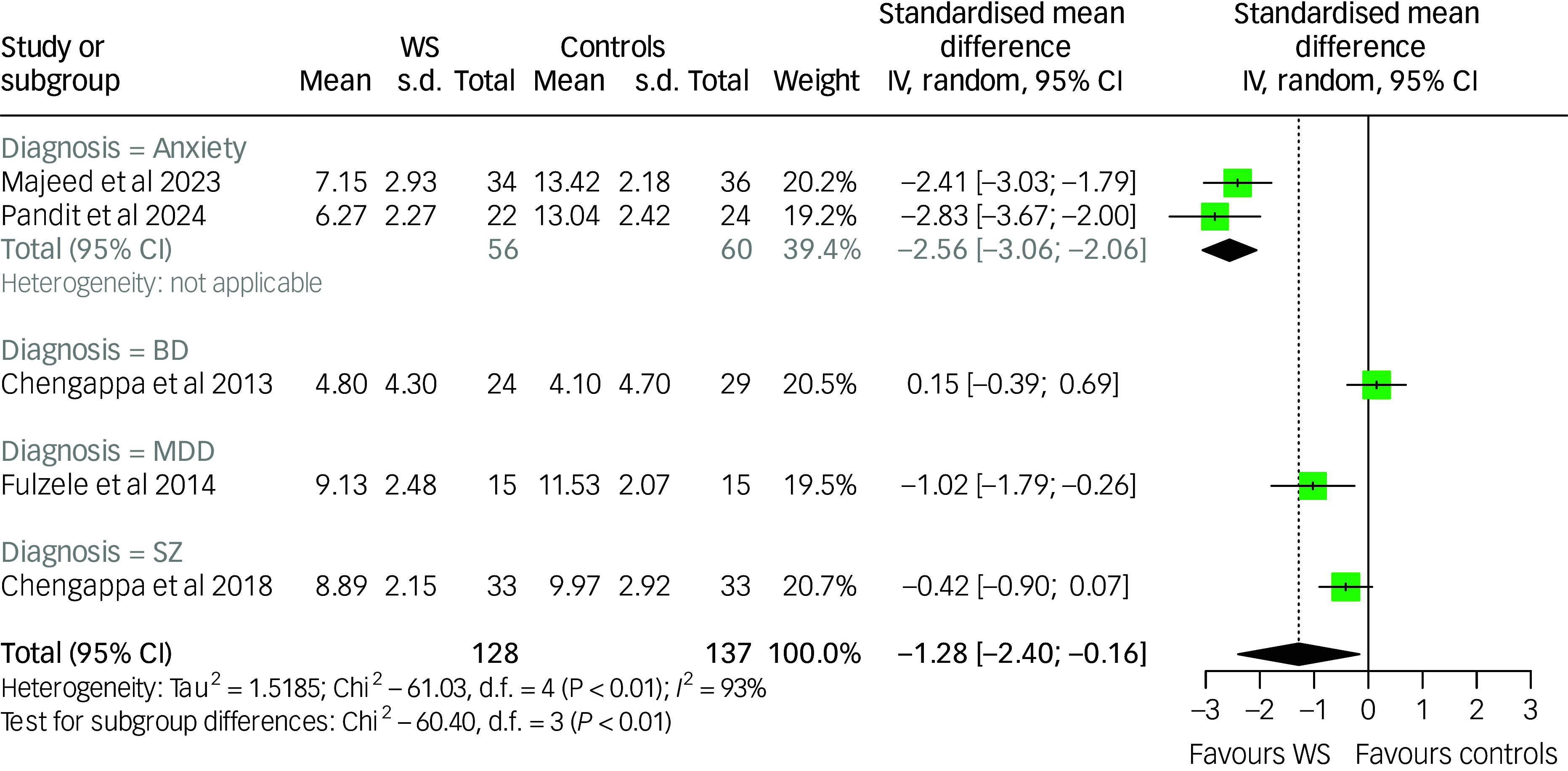
Forest plot of depression among *Withania somnifera* and control groups: overall and by diagnostic subgroup. BD, bipolar disorder; IV, inverse variance; MDD, major depressive disorder; SZ, schizophrenia; WS, *Withania somnifera*. The measures of depression across the studies were scaled as lower scores indicate lower depression symptoms. Reference to the included studies (in alphabetical order): Chengappa et al, 2013^42^; Chengappa et al, 2018^43^; Fulzele et al, 2014^47^; Majeed et al, 2023^52^; Pandit et al, 2024.^53^

The meta-analysis of the effect of *Withania somnifera* on depression symptoms included five studies, and specifically for one of these the outcome data were extracted from a secondary publication on the same sample.^54^ The pooled estimate of the treatment effect favoured *Withania somnifera* (SMD: −1.28 (95% CI: −2.40; −0.16])), with high between-study heterogeneity (*I* ^2^: 93%; *p*-value <0.001). While generally recommended with a minimum of ten studies, we however conducted an exploratory subgroup analysis based on participants’ diagnoses to investigate potential sources of heterogeneity. The test for subgroup differences was statistically significant (*p*-value <0.001), with the largest effect of *Withania somnifera* on depression symptoms observed in studies involving participants with anxiety, followed by those with MDD. No significant effect was found in the single studies involving participants with bipolar disorder and schizophrenia. These results should be interpreted cautiously due to the limited number of studies within each subgroup. The results are summarised in Fig. 4.

Due to the limited number of studies, we did not conduct a test for publication bias and meta-regression.

Leave-one-out analysis showed that by excluding the study from Chengappa et al,^42^ the pooled estimate favouring *Withania somnifera* reached statistical significance (SMD: −1.28 (95% CI: −2.45; -0.10)), with high heterogeneity (*I* ^2^: 91%). In addition, by excluding the study from Majeed et al,^52^ there was a marked decrease in the heterogeneity (*I* ^2^: 71%) and in the effect size (SMD: −0.39 (95% CI: −1.01; 0.24)). The results of the leave-one-out analysis are available in Supplementary Table 7.

**Fig. 5:**
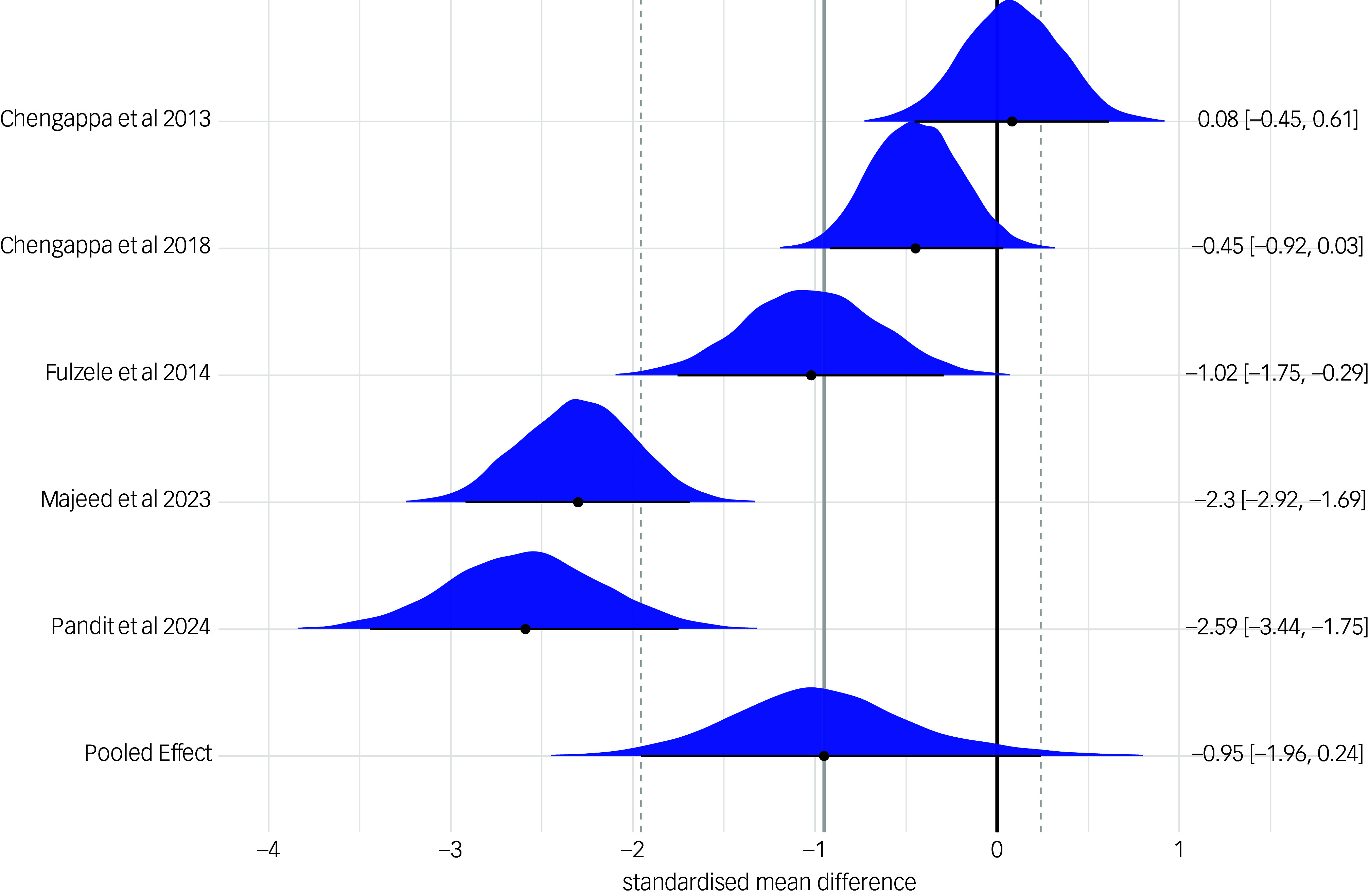
Bayesian forest plot of study-level and pooled posterior estimates of *Withania somnifera* efficacy on depression. The measures of depression across the studies were scaled as lower scores indicate lower depression symptoms. Reference to the included studies (in alphabetical order): Chengappa et al, 2013^42^; Chengappa et al, 2018^43^; Fulzele et al, 2014^47^; Majeed et al, 2023^52^; Pandit et al, 2024.^53^

To further examine the robustness of the findings in a context with high heterogeneity, we performed Bayesian hierarchical meta-analysis using weakly informative priors (i.e. normal(0,1) for the intercept, cauchy(0,0.5) and exponential(1) for τ). Both models converged at 4000 iterations and produced similar findings in terms of posterior distribution of τ and the overall treatment effect (see Supplementary Figure 3; ∆ELPD: −0.3). Given its conservative features, the model using a cauchy(0,0.5) prior was retained as the primary analysis. This model yielded a non-statistically significant treatment effect (SMD: −0.95 (95% CrI: −1.96; 0.24)) and high between-study heterogeneity (τ: 1.29 (95% CrI: 0.62; 2.66)). The results of the Bayesian meta-analysis are displayed in Fig. 5.

### Effect of *Withania somnifera* on sleep quality

**Fig. 6:**
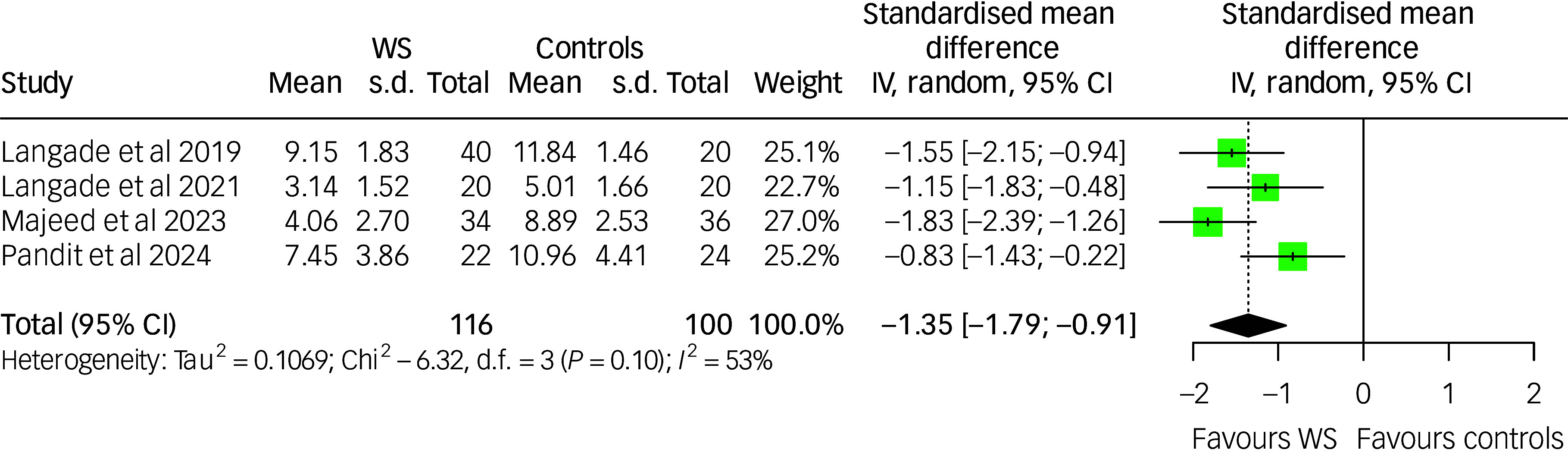
Forest plot of sleep quality among *Withania somnifera* and control groups. IV: inverse variance; WS, *Withania somnifera*. The measures of sleep quality across the studies were scaled as lower scores indicate better sleep quality. Reference to the included studies (in alphabetical order): Langade et al, 2019^50^; Langade et al 2021^51^; Majeed et al, 2023^52^; Pandit et al, 2024.^53^

The meta-analysis of the effect of *Withania somnifera* on sleep included four studies. The pooled estimate favoured *Withania somnifera* (SMD: −1.35 (95% CI: −1.79; −0.91)) with moderate between-study heterogeneity (*I* ^2^: 53%, *p*-value: 0.097). The results are presented in Fig. 6.

Due to the limited number of studies included in the meta-analysis, sensitivity analyses could not be performed.

### Effect of *Withania somnifera* on other mental health symptoms

**Fig. 7:**
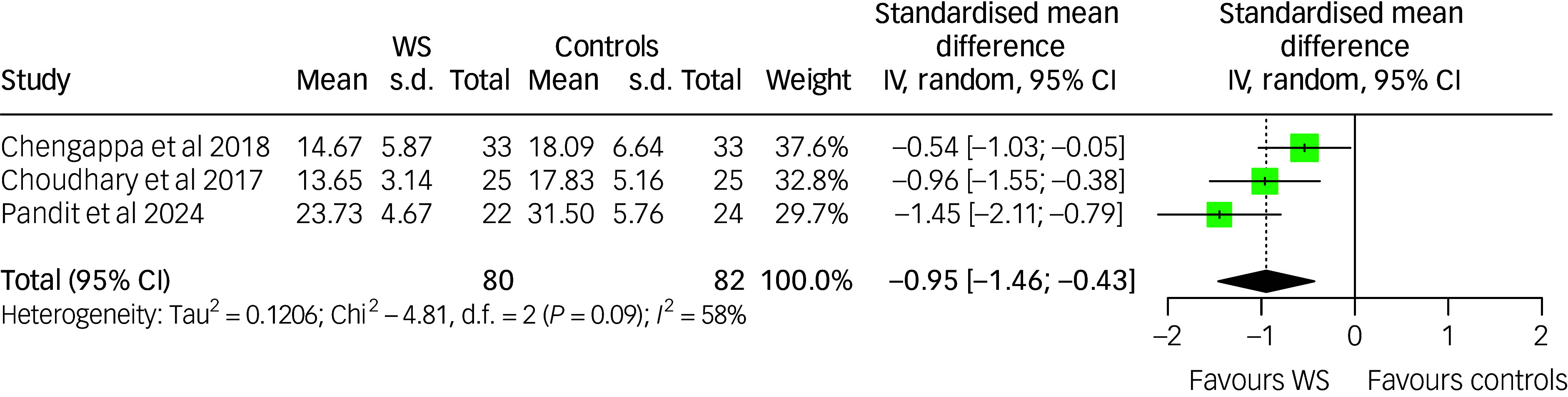
Forest plot of perceived stress among *Withania somnifera* and control groups. IV: inverse variance; WS: *Withania somnifera*. The measures of stress across the studies were scaled as lower scores indicate lower perceived stress. Reference to the included studies (in alphabetical order): Chengappa et al, 2018^43^; Choudhary et al, 2017^44^; Pandit et al, 2024.^53^

Four other outcome measures were reported in the included studies. These consisted of schizophrenia symptoms, mania symptoms, OCD symptoms and perceived stress. Except for perceived stress, each outcome was reported by only one study, precluding meta-analysis. The meta-analysis for perceived stress was based on three studies, indicating a significant effect in favour of *Withania somnifera* (SMD: −0.95 (95% CI: −1.46; −0.43)), with moderate between-study heterogeneity (*I* ^2^: 58%, *p*-value: 0.090). The results are shown in Fig. 7.

### Analysis of safety and tolerability

**Fig. 8:**
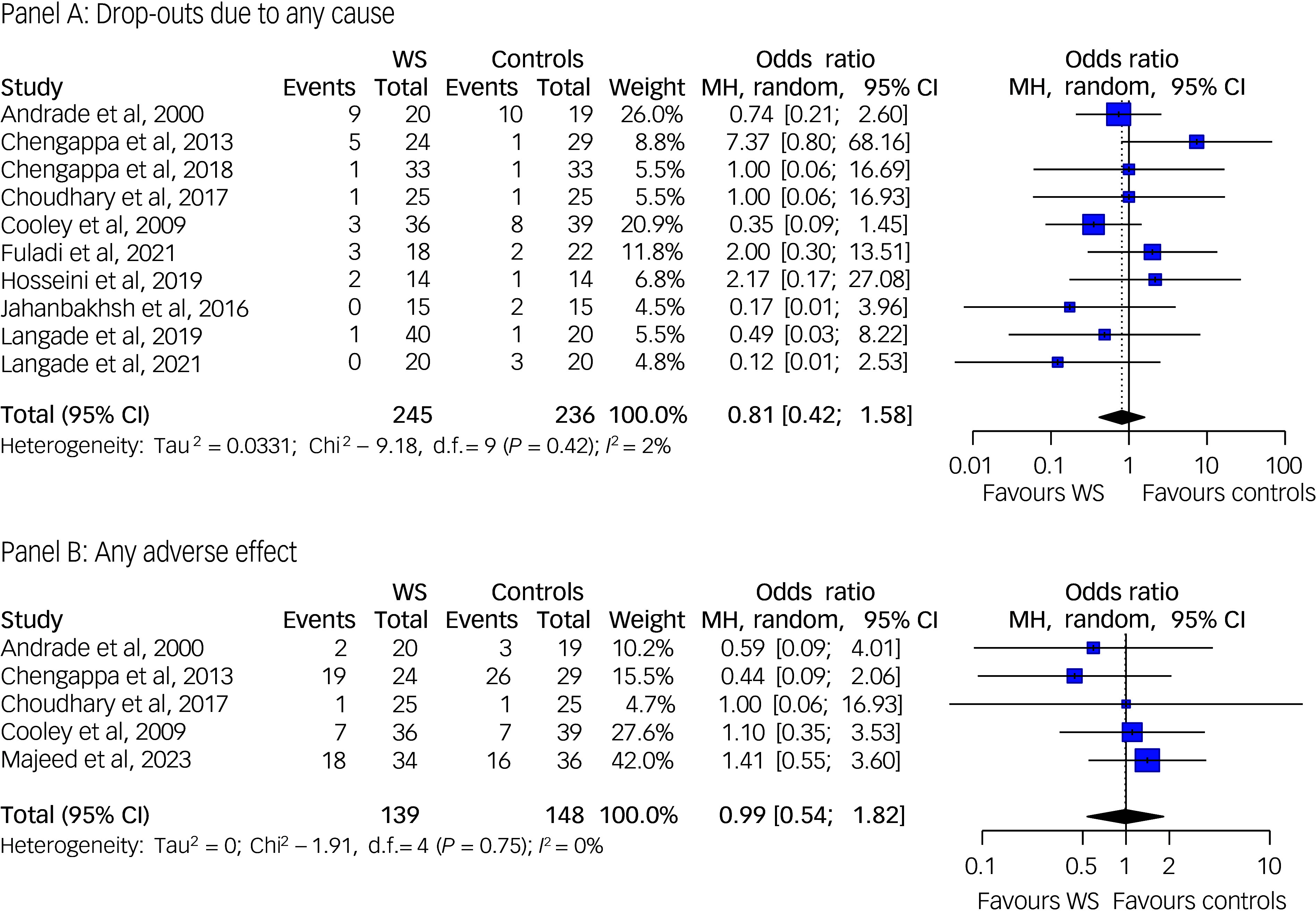
Forest plots of drop-out due to any cause (Panel A) and any adverse effect (Panel B) among *Withania somnifera* and control groups. MH: Mantel-Haenszel; WS: *Withania somnifera*. Lower rates indicate better safety/tolerability. Reference to the included studies (in alphabetical order): Andrade et al, 2000^41^; Chengappa et al, 2013^42^; Chengappa et al, 2018^43^; Choudhary et al, 2017^44^; Cooley et al, 2009^45^; Fuladi et al, 2021^46^; Hosseini et al, 2019^48^; Jahanbakhsh et al, 2016^17^; Langade et al, 2019^50^; Langade et al 2021^51^; Majeed et al, 2023.^52^

The analysis of the safety and tolerability of *Withania somnifera* treatment was made by assessing the rates of adverse events, drop-out due to any cause and drop out due to death or serious adverse events across the experimental and control groups. There were four studies^46–49^ not reporting information on the number of treatment’s adverse effects across the trial’s arms, although these stated in the respective papers that there were no significant differences between the experimental and control groups. Additionally, two studies^47,49^ did not report information on the number of drop-outs during the trial. Notably, no death or drop-out due to severe treatment adverse effects occurred both in the experimental and in the control groups of all the included trials. The meta-analysis of the drop-out due to any cause included ten studies, and the meta-analysis of the rate of any adverse event was based on five studies. Our investigation found similar rates of drop-out due to any cause and of any adverse effect across the experimental and control groups, supporting no significant differences between *Withania somnifera* and the placebo in terms of safety and tolerability (odds ratio 0.81 (95% CI: 0.42; 1.58) and odds ratio 0.99 (95% CI: 0.54; 1.82) for the rate of drop-out due to any cause and any adverse effect, respectively), with no evidence of between-study heterogeneity. The most reported adverse events were gastro-intestinal disturbances (e.g. diarrhoea or dyspepsia), which were transitory and not severe. The results are displayed in Fig. 8.

### GRADE of the evidence

A detailed summary on the risk of bias in all 14 trials is reported in Supplementary Figs 4 and 5, along with an assessment of the quality of the evidence (see Supplementary Table 8). In the GRADE system, the evidence from RCTs is initially set to high; there are then criteria that can be used either to downgrade or upgrade (see further information in the Supplementary Material). The quality of the evidence ranged from very low for depression symptoms to low for anxiety symptoms, sleep quality and stress. The main reasons for downgrading the certainty of evidence were the limited number of studies contributing to each pooled estimate, the high level of heterogeneity between the studies and bias threats related to the blinding procedures.

## Discussion

This systematic review set out to investigate the efficacy and safety of the treatment with *Withania somnifera* on people with mental disorders. Specifically, we synthesised evidence on the effect of *Withania somnifera* on anxiety and depression symptoms, while also exploring its effects on sleep quality, schizophrenia, mania, OCD and perceived stress. Our investigation points out a statistically significant effect of *Withania somnifera* in improving anxiety, sleep quality and stress. The effect on anxiety symptoms should be interpreted considering high between-study heterogeneity, and was limited on the other mental health outcomes by the small number of studies contributing to each pooled estimate. In spite of this, the observed anxiolytic effect aligns with previous findings on both clinical and healthy populations.^4,12,55^ However, our study is the largest meta-analysis on *Withania somnifera* in clinical settings. Interestingly, the observed anxiolytic effect of *Withania somnifera* extends beyond anxiety disorder populations, having also been observed in individuals with ADHD, depression and insomnia.^48,50–52^ Although only one trial in ADHD and one trial in depression investigated the effect of *Withania somnifera* on anxiety symptoms, its broad pharmacological profile, modulating GABAergic, cholinergic and anti-inflammatory pathways,^1,9,10^ could underlie its trans-diagnostic anxiolytic effects. However, it is interesting to note that the study from Chengappa et al,^42^ which was conducted on patients with bipolar disorder in clinical remission, provided estimates crossing the line of no effect for both anxiety and depression symptoms. Similarly, the other study from Chengappa et al,^43^ performed on people with acute exacerbation of schizophrenia, did not find evidence supporting the efficacy of the treatment on depressive symptoms of participants. Finally, the study from Fulzele et al^47^ that was performed on participants with mild to moderate depression also reported a small, yet significant, effect supporting the anxiolytic and antidepressant effect of *Withania somnifera*. It could be argued that in both clinically severe or clinically stable patients, anxiety and depressive symptoms are very unlikely to improve any further with *Withania somnifera*.

We found a dose–response relationship for the anxiolytic effect, although caution is warranted as doses ranged widely across studies (from 10 to 12 000 mg/day) and this evidence was markedly influenced by one study which administered an exceptionally high dose of *Withania somnifera* (12 000 mg daily).^49^ However, our Bayesian meta-analysis suggested no statistically significant advantage of doses above 600 mg/day, indicating that this median dose may represent an optimal balance of efficacy and tolerability. It is important to acknowledge that, beyond the dose, *Withania somnifera* extracts exhibit differences in the bioactives composition influencing specific neural circuits, neurotransmitters, inflammatory responses, redox mechanisms and immune systems, all of which are purportedly relevant to mental disorders. Despite challenges in obtaining detailed information on the composition of *Withania somnifera* extracts used in the included trials, our investigation found no significant interaction of dose and minimum withanolides concentration on the anxiolytic effect. Previous studies on the pharmacokinetic features of *Withania somnifera* reported a short half-life, requiring two or three daily oral administrations to reach adequate bioavailability.^6,56^ Speculatively, these characteristics may contribute to dose-related effects and could limit the sustained efficacy of *Withania somnifera* if dosing is suboptimal or inconsistent. However, the multitude of bioactives in *Withania somnifera* extracts and the inconsistent reporting of these across the trials, limit considerations of *Withania somnifera* pharmacokinetics and pharmacodynamics. Additionally, most of the studies on the pharmacological and safety profile of *Withania somnifera* were performed on doses around 500 mg/day, therefore the use of higher doses of *Withania somnifera* should be further investigated, especially in terms of safety when administered with other treatments.

The frequentist meta-analysis for depressive symptoms yielded a statistically significant pooled effect favouring *Withania somnifera*, although with high between-study heterogeneity. Importantly, the effect was not confirmed in the Bayesian hierarchical approach, emphasising uncertainty under different assumptions. This discrepancy indicates that the evidence for the antidepressant effect of *Withania somnifera* is less robust and more likely influenced by small-studies effects, between-study heterogeneity or confounding factors and therefore should be interpreted with caution. Notably, by looking at the contribution of the individual studies, leave-one-out analysis showed a potentially enhanced antidepressant effect in individuals with concurrent anxiety and stress, providing hints for future trials. Additionally, the study from Majeed et al^52^ which administered a *Withania somnifera* extract supplemented with piperine – a compound with antidepressant-like properties^57,58^ – showed a particularly large antidepressant effect.

Although the meta-analyses of sleep quality and stress were based on a small number of studies limiting the certainty of the evidence, the pooled effects were statistically significant in favour of *Withania somnifera*, warranting further investigation of *Withania somnifera* in stress and insomnia treatment in large and high-quality RCTs. The beneficial effect of *Withania somnifera* on sleep quality and stress confirmed previous research findings both among clinical samples and healthy volunteers.^12,13,59^ These effects, substantiated by the found anxiolytic properties, reinforce *Withania somnifera* adaptogen potential.

The meta-analysis was precluded for mania, OCD and schizophrenia symptoms as each was assessed in only one trial. However, these individual studies reported a reduction in schizophrenia symptoms for *Withania somnifera* used as an add-on to antipsychotics, whereas no significant effects were observed for mania or OCD symptoms. Similar results were obtained for other add-on treatments for schizophrenia, such as sarcosine and antioxidants,^60,61^ which share some similarities with *Withania somnifera*, warranting further investigations on *Withania somnifera* as a potential treatment option with a good tolerability.

Finally, in terms of safety and tolerability, *Withania somnifera* treatment appears to be safe and well-accepted by patients, as supported by the similar rate of drop-out due to any cause and of adverse effects across the treatment and control arms. The reported adverse events were generally mild, transitory and did not require treatment interruption.

### Limitations

The results of this study should be interpreted considering its limitations.

First, the relatively small number of studies included in the final selection limited the possibility to perform robust meta-analytic investigations. This limitation resulted in imprecision, evident in large confidence intervals or confidence intervals crossing the line of no-effect and susceptibility to effect of outlier studies. In addition, three out of the four meta-analyses performed the number of studies included was less than ten, falling short of the threshold required for robust testing of publication bias.^27^ Further, for mania, OCD and schizophrenia there was only one trial available, significantly limiting any possibility to make suggestions on the use of *Withania somnifera* in these specific disorders. Second, *Withania somnifera* is a treatment from the Ayurvedic medicine tradition. However, the identification of studies was conducted across databases in the biomedical sciences, which may have limited coverage of traditional and alternative medicine reports. This limitation may challenge the comprehensiveness of the search strategy. Third, there was substantial between-study heterogeneity, compounded by studies providing exceptionally outlier estimates, posing challenges for a nuanced investigation of the source of heterogeneity. Fourth, the risk of bias assessment found threats related to allocation and blinding procedures, reflected in the low to very low grading of the evidence. Therefore, these findings should be interpreted with caution and confirmed in future high-quality trials. Fifth, some studies did not report information on some participants’ characteristics, *Withania somnifera* extract composition, concurrent treatments or the adverse events of treatment. Stricter adherence to the consolidated standards of reporting trials is needed. Finally, the selected studies were all performed in India, Iran or North America. This geographical limitation potentially influences the generalisability of the findings.

### Implications for research and practice

The results of this research highlight the therapeutic potential of *Withania somnifera*, whose main benefits were observed in the treatment of anxiety, which is one of the most common mental disorders.^62^ Despite a large effect size observed on anxiety symptoms, the certainty of the evidence was rated low mainly due to high between-study heterogeneity.

Sleep disorders and depressive symptoms are also rather common in the general population even as transient symptoms and display a close correlation with anxiety disorders.^63,64^ This correlation extends beyond epidemiological observations, delving into shared neurobiological underpinnings which could be effectively targeted by the *Withania somnifera* treatment.^65,66^ The improvement in anxiety and stress-related symptoms could account for a better ability to regulate emotions in response to adverse stimuli, consistent with the suggested adaptogen effect of *Withania somnifera*.^67^ In future, it could be rational to investigate prospective studies of whether *Withania somnifera* can be used in individuals with high levels of stress to prevent them from transitioning to anxiety disorders.

The broad pharmacological profile could be useful in other disorders, like depression, or conditions with a strong link to environmental risk factors, such as stress and burnout. Furthermore, the reported enhancements in cognition and the anti-inflammatory properties of *Withania somnifera* add layers to its potential applicability in other psychiatric conditions.^14,20,68^ Interestingly, *Withania somnifera* also showed elsewhere the positive impact on diabetes management, anti-ischaemic properties and effects on sexual function, comorbidities often associated with psychiatric disorders.^14,69,70^ Future trials could investigate the benefit of *Withania somnifera* supplementation for individuals with mental disorders, especially those vulnerable to developing these comorbidities.

While the results of this study suggest a favourable safety profile of *Withania somnifera*, in agreement with previous research,^7,56,71^ the need for cautious observation persists. Standardisation of*Withania somnifera* titles in commercial preparation should be pursued to ensure that users receive the expected benefits and to support accurate assessments of its pharmacological effects in research. In addition, large-scale investigations, with a specific focus on concomitant use with other medications and potential interactions, are needed. The safety and tolerability aspects, derived from a synthesis of clinical and preclinical evidence, should guide future randomised trials. These studies should adhere to robust therapeutic and safety standards, conducted on a larger scale, to improve understanding of *Withania somnifera*’s potential in psychiatric care.

In conclusion, our study provided evidence for the clinical effectiveness of at least 600 mg/day of *Withania somnifera* in reducing anxiety symptoms. Despite the large anxiolytic effect size found, the high between-study heterogeneity warrants cautious interpretation. Additionally, a positive effect of *Withania somnifera* was found also on sleep quality and perceived stress, although caution is advised in these findings due to the limited data available. Future research should prioritise exploring *Withania somnifera*’s role in anxiety, depression and insomnia treatment, considering its promising trends. Overall, although *Withania somnifera* showed potential in psychiatric care, more, thorough, studies are crucial to define its therapeutic boundaries and optimise its clinical utility.

## Supporting information

Marchi et al. supplementary material Marchi et al. supplementary material

## Data Availability

The codes for reproducing the dataset and the analyses can be accessed here: https://github.com/MattiaMarchi/Withania-somnifera_Ashwagandha_-mental-illness.

## Supplementary material

The supplementary material is available online at https://doi.org/10.1192/bjo.2025.10885
