# Supplementary material for: The effect of Withania somnifera (Ashwagandha) on mental health symptoms in individuals with mental disorders: systematic review and meta-analysis
Source: BJPsych Open. 2025 Oct 27;11(6):e260. doi: 10.1192/bjo.2025.10885 (PMC12569615; doi:10.1192/bjo.2025.10885)
Supplement: Marchi et al. supplementary material [file S2056472425108855sup001.docx]

**Online Appendix - Supplementary Materials**

Supplementary Table 1: Search strategy and number of hits per database (current to 15^th^ May 2025).

| **Platform**  ***(Database)*** | **String** | **Number of records** |
| --- | --- | --- |
| PubMed  *(Medline)* | ((("Ashwagandha" [Supplementary Concept]) OR ("Withania"[Mesh]) OR ("Ashwagandha") OR ("Withania") OR ("Somnifera")) AND ("Mental Disorders"[Mesh])) AND (("Randomized Controlled Trial" [Publication Type]) OR ("Randomized Controlled Trials as Topic"[Mesh])) | 20 |
| Scopus  *(Scopus)* | ( ( TITLE-ABS-KEY ( ashwagandha ) OR TITLE-ABS-KEY ( withania ) OR TITLE-ABS-KEY ( withania AND somnifera ) ) ) AND ( ( TITLE-ABS-KEY ( mental AND disorder* ) OR TITLE-ABS-KEY ( mental AND illenss* ) OR TITLE-ABS-KEY ( mental AND disease* ) ) ) AND ( TITLE-ABS-KEY ( randomized AND controlled AND trial* ) ) | 29 |
| EMBASE, PICO search with exploding terms  *(EMBASE)* | **Population:** ('mental disease'/exp OR 'abnormal mental state' OR 'disease, mental' OR 'diseased mental state' OR 'disorder, mental' OR 'disordered mental state' OR 'disturbed mental state' OR 'illness, mental' OR 'insanity' OR 'mental abnormality' OR 'mental change' OR 'mental confusion' OR 'mental defect' OR 'mental disease' OR 'mental disorder' OR 'mental disorders' OR 'mental disorders diagnosed in childhood' OR 'mental disturbance' OR 'mental illness' OR 'mental insufficiency' OR 'mental symptom' OR 'mentally ill' OR 'neurodevelopmental disorder' OR 'neurodevelopmental disorders' OR 'neuropsychiatric disease' OR 'neuropsychiatric diseases' OR 'neuropsychiatric disorder' OR 'neuropsychiatric disorders' OR 'psychiatric disease' OR 'psychiatric disorder' OR 'psychiatric illness' OR 'psychiatric symptom' OR 'psychic disease' OR 'psychic disorder' OR 'psychic disturbance' OR 'psychologic disorder' OR 'psychologic disturbance' OR 'psychological disorder' OR 'psychological disturbance' OR 'psychopathology')  AND  **Intervention:** ('withania somnifera'/exp OR 'withania somnifera' OR 'ashwagandha' OR 'indian ginseng' OR 'whithania somnifera')  AND  **Study design:** ('randomized controlled trial'/exp OR 'controlled trial, randomized' OR 'randomised controlled study' OR 'randomised controlled trial' OR 'randomized controlled study' OR 'randomized controlled trial' OR 'trial, randomized controlled') in ti,ab,kw | 68 |
| EBSCO  *(PsycINFO)* | (ashwagandha OR withania somnifera OR withania) AND (DE "Mental Disorders" OR DE "Affective Disorders" OR DE "Anxiety Disorders" OR DE "Bipolar Disorder" OR DE "Borderline States" OR DE "Chronic Mental Illness" OR DE "Dissociative Disorders" OR DE "Eating Disorders" OR DE "Gender Dysphoria" OR DE "Mental Disorders due to General Medical Conditions" OR DE "Neurocognitive Disorders" OR DE "Neurodevelopmental Disorders" OR DE "Neurosis" OR DE "Paraphilias" OR DE "Personality Disorders" OR DE "Psychosis" OR DE "Serious Mental Illness" OR DE "Sleep Wake Disorders" OR DE "Somatoform Disorders" OR DE "Stress and Trauma Related Disorders" OR DE "Substance Related and Addictive Disorders" OR DE "Thought Disorders") AND (DE "Randomized Controlled Trials" OR DE "Clinical Trials" OR DE "Randomized Clinical Trials") | 3 |
| EBSCO  *(CINAHL)* | (withania somnifera OR withania OR ashwagandha) AND (MH "Mental Disorders+") AND ((MH "Clinical Trials+") OR (MH "Randomized Controlled Trials+")) | 6 |
| CENTRAL  *(ICTRP & CT.gov)* | (("ashwagandha"):ti,ab,kw OR ("Withania somnifera"):ti,ab,kw) AND (mental illness):ti,ab,kw AND ("randomized controlled trial"):ti,ab,kw | 260  (237+23) |

Legend: the first column lists the name of each platform searched, with the database(s) included shown in parentheses.

Supplementary Table 2: List of the studies excluded after analysis of full text and reason for exclusion

| **Author, year** | **Reason for exclusion** |
| --- | --- |
| Agnihotri et al., 2013 ^1^ | The outcome is not mental health symptoms. |
| Baker et al., 2022 ^2^ | Qualitative study without quantitative outcome measures on a population of healthy college students. |
| Choudhary et al., 2017 ^3^ | The population is people with dementia or mild cognitive impairment. |
| Dimpfel et al., 2020 ^4^ | The population is people with dementia or mild cognitive impairment, and the outcome is not mental health symptoms. |
| Gannon et al., 2019 ^5^ | Multiple publication of data from the same sample of Chengappa et al., 2018. |
| NCT03437668 ^6^ | Incomplete trial with the following status: “Terminated: Unable to reach enrollment target due to COVID”. Results posted but work unpublished. |

**Reference list of the studies excluded after full text analysis**

1 Agnihotri AP, Sontakke SD, Thawani VR, Saoji A, Goswami VSS. Effects of Withania somnifera in patients of schizophrenia: a randomized, double blind, placebo controlled pilot trial study. *Indian J Pharmacol* 2013; **45**: 417–8.

2 Baker C, Kirby JB, O’Connor J, Lindsay KG, Hutchins A, Harris M. The Perceived Impact of Ashwagandha on Stress, Sleep Quality, Energy, and Mental Clarity for College Students: Qualitative Analysis of a Double-Blind Randomized Control Trial. *J MEDICINAL FOOD* 2022; **25**: 1095–101.

3 Choudhary D, Bhattacharyya S, Bose S. Efficacy and Safety of Ashwagandha (Withania somnifera (L.) Dunal) Root Extract in Improving Memory and Cognitive Functions. *J Diet Suppl* 2017; **14**: 599–612.

4 Dimpfel W, Schombert L, Keplinger-Dimpfel IK, Panossian A. Effects of an adaptogenic extract on electrical activity of the brain in elderly subjects with mild cognitive impairment: A randomized, double-blind, placebo-controlled, two-armed cross-over study. *Pharmaceuticals* 2020; **13**. doi:10.3390/ph13030045.

5 Gannon JM, Brar J, Rai A, Chengappa KNR. Effects of a standardized extract of Withania somnifera (Ashwagandha) on depression and anxiety symptoms in persons with schizophrenia participating in a randomized, placebo-controlled clinical trial. *Ann Clin Psychiatry* 2019; **31**: 123–9.

6 NCT03437668. Adjunctive Withania Somnifera (Ashwagandha) for Persistent Symptoms in People With Schizophrenia. *https://clinicaltrials.gov/ct2/show/NCT03437668* 2018. (https://www.cochranelibrary.com/central/doi/10.1002/central/CN-01523105/full).

Supplementary Table 3: Composition of Withania somnifera extract used in each study.

| **Author, year**  **(Trial ID)** | **Treatment** | **Constituents** | **Branded name** | **Part of the plant** |
| --- | --- | --- | --- | --- |
| Andrade et al, 2000  (NR) | WS 500 mg po daily | NR | NR | NR |
| Chengappa et al, 2013  (NCT00761761) | WS 500 mg po daily | ≥8% whitanolides; 32% oligosaccharides; ≥2% withaferin A | Sensoril | Leaf and root |
| Chengappa et al, 2018  (NCT01793935) | WS 1000 mg po daily | ≥8% whitanolides; 32% oligosaccharides; ≥2% withaferin A | Sensoril | Leaf and root |
| Choudhary et al, 2017  (NR) | WS 600 mg po daily | ≥5% whitanolides | KSM-66 Ashwagandha | Root |
| Cooley et al, 2009  (ISRCTN78958974) | WS 600 mg po daily | ≥1.5% whitanolides | Swiss-Ashwagandha | Root |
| Fuladi et al, 2021  (IRCT20180615040105N1) | WS 1000 mg po daily | NR | NR | Root |
| Fulzele et al, 2014  (NR) | WS po daily (dose NR) | NR | NR | NR |
| Hosseini et al, 2019  (IRCT201506215280N18) | WS 10 mg po daily | NR | NR | Root |
| Jahanbakhsh et al, 2016  (IRCT2015070523079N1) | WS 1000 mg po daily | NR | NR | Root |
| Khyati et al, 2013  (NR) | WS 12000 mg po daily | NR | NR | Root |
| Langade et al, 2019  (NR) | WS 600 mg po daily | ≥5% whitanolides | KSM-66 Ashwagandha | Root |
| Langade et al, 2021  (CTRI/2019/03/018074) | WS 600 mg po daily | ≥5% whitanolides | KSM-66 Ashwagandha | Root |
| Majeed et al, 2023  (CTRI/2022/05/042640) | WS 500 mg po daily | ≥2.5% whitanolides | Linn-Shagandha | Root |
| Pandit et al, 2024  (CTRI/2019/11/022100) | WS 500 mg po daily | ≥8% whitanolides; 32% oligosaccharides; ≥2% withaferin A | Sensoril | Leaf and root |

Abbreviations (in alphabetical order): mg: milligram; NR: information not reported; po: per os; WS: Withania somnifera.

Supplementary Table 4: outcome measures used in each study

| **Author, year**  **(Trial ID)** | **Anxiety** | **Depression** | **Sleep quality** | **Other outcomes** |
| --- | --- | --- | --- | --- |
| Andrade et al, 2000  (NR) | HAM-A | NA | NA | NA |
| Chengappa et al, 2013  (NCT00761761) | HAM-A | MADRS | NA | YMRS |
| Chengappa et al, 2018  (NCT01793935) | NA | Clustered items from PANSS | NA | PANSS, PSS |
| Choudhary et al, 2017  (NR) | NA | NA | NA | PSS |
| Cooley et al, 2009  (ISRCTN78958974) | BAI | NA | NA | NA |
| Fuladi et al, 2021  (IRCT20180615040105N1) | HAM-A | NA | NA | NA |
| Fulzele et al, 2014  (NR) | NA | HDRS | NA | NA |
| Hosseini et al, 2019  (IRCT201506215280N18) | RCMA | NA | NA | NA |
| Jahanbakhsh et al, 2016  (IRCT2015070523079N1) | NA | NA | NA | Y-BOCS |
| Khyati et al, 2013  (NR) | HAM-A | NA | NA | NA |
| Langade et al, 2019  (NR) | HAM-A | NA | PSQI | NA |
| Langade et al, 2021  (CTRI/2019/03/018074) | HAM-A | NA | PSQI | NA |
| Majeed et al, 2023  (CTRI/2022/05/042640) | HAM-A | HDRS | GSQS | NA |
| Pandit et al, 2024  (CTRI/2019/11/022100) | HAM-A | HDRS | PSQI | PSS |

Abbreviations (in alphabetical order): BAI: Beck anxiety inventory; GSQS: Groningen sleep quality scale; HAM-A: Hamilton anxiety rating scale; HDRS: Hamilton depression rating scale; MADRS: Montgomery-Asberg depression rating scale; NA: not applicable; PANSS: positive and negative syndrome scale; PSQI: Pittsburgh sleep quality index; PSS: perceived stress scale; RCMA: revised children's manifest anxiety scale; Y-BOCS: Yale-Brown obsessive compulsive scale; YMRS: young mania rating scale.

Supplementary Figure 1: Funnel plot of publication bias for the treatment effect on anxiety.

Supplementary Table 5: Leave-one-out analysis of anxiety among Withania somnifera and control groups.

| **Study ID** | **SMD (95% CI)** | **p-value** | **I^2^** |
| --- | --- | --- | --- |
| Andrade et al, 2000 | -1.73 (-2.88; -0.57) | 0.003 | 97% |
| Chengappa et al, 2013 | -1.78 (-2.90; -0.67) | 0.002 | 96% |
| Cooley et al, 2009 | -1.75 (-2.89; -0.61) | 0.003 | 96% |
| Fuladi et al, 2021 | -1.72 (-2.88; -0.57) | 0.003 | 97% |
| Hosseini et al, 2019 | -1.62 (-2.79; -0.44) | 0.007 | 97% |
| Khyati et al, 2013 | -1.13 (-1.65; -0.60) | <0.001 | 85% |
| Langade et al, 2019 | -1.70 (-2.87; -0.54) | 0.004 | 97% |
| Langade et al, 2021 | -1.68 (-2.85; -0.51) | 0.005 | 97% |
| Majeed et al, 2023 | -1.52 (-2.67; -0.37) | 0.010 | 97% |
| Pandit et al, 2024 | -1.56 (-2.72; -0.39) | 0.009 | 97% |

Abbreviations (in alphabetical order): SMD: standardized mean difference; 95%CI: 95% confidence interval.

Supplementary Figure 2: posterior distributions of between-study heterogeneity (τ) under different priors for anxiety meta-analysis.

Supplementary Figure 3: posterior distributions of between-study heterogeneity (τ) under different priors for depression meta-analysis.

Supplementary Table 6: Results of univariable meta-regression analysis on the effect of Whitania somnifera on anxiety.

| **Variable(s)** | **B (95% CI)** | **p-value** |
| --- | --- | --- |
| Age | 0.033 (-0.014; 0.080) | 0.164 |
| % Females | 0.033 (-0.006; 0.072) | 0.097 |
| Country:  Canada  USA  Iran  India | -0.473 (-4.02; 3.07)  0.318 (-4.71; 5.34)  -0.703 (-5.07; 3.66)  -1.74 (-5.58; 2.10) | 0.794  0.901  0.752  0.374 |
| WS dose (mg) | -0.0004 (-0.0006; -0.0003) | <0.001 |
| Dose*Withanolides% | 0.0001 (-0.0006; 0.0008) | 0.807 |
| Duration (weeks) | -0.023 (-0.537; 0.492) | 0.931 |
| Use:  Monotherapy  Add-on | -0.456 (-1.54; 0.626)  -0.753 (-1.47; -0.038) | 0.409  0.039 |

Abbreviations (in alphabetical order): B: unstandardized linear regression coefficient; NA: information not available; WS: Whitania somnifera; 95% CI: 95% confidence intervals.

Supplementary Table 7: Leave-one-out analysis of depression among Withania somnifera and control groups.

| **Study ID** | **SMD (95% CI)** | **p-value** | **I^2^** |
| --- | --- | --- | --- |
| Chengappa et al, 2013 | -1.65 (-2.77; -0.53) | 0.004 | 92% |
| Chengappa et al, 2018 | -1.51 (-2.86; -0.17) | 0.027 | 94% |
| Fulzele et al, 2014 | -1.36 (-2.79; 0.08) | 0.063 | 96% |
| Majeed et al, 2023 | -1.00 (-2.25; 0.25) | 0.116 | 94% |
| Pandit et al, 2024 | -0.91 (-2.00; 0.17) | 0.099 | 93% |

Abbreviations (in alphabetical order): SMD: standardized mean difference; 95%CI: 95% confidence interval.

Supplementary Figure 4: Risk of bias graph showing review authors' judgements about each risk of bias item presented as percentages across all included studies.

Supplementary Figure 5: Risk of bias summary showing review authors' judgements about each risk of bias item for each included study.

Supplementary Table 8: GRADE evidence summary for each outcome

| **Certainty assessment** | | | | | | | **N of patients** | | **Effect** | **Certainty** | **Importance** |
| --- | --- | --- | --- | --- | --- | --- | --- | --- | --- | --- | --- |
| **N of studies** | **Study design** | **Risk of bias** | **Inconsistency** | **Indirectness** | **Imprecision** | **Other considerations** | **WS** | **Controls** | **SMD**  **(95% CI)** |  |  |
| *Anxiety* | | | | | | | | | | | |
| 10 | RCT | Serious | Serious | Not serious | Not serious | Not serious | 272 | 265 | -1.62  (-2.66; -0.57) | ++??  LOW | Important |
| *Depression* | | | | | | | | | | | |
| 5 | RCT | Serious | Serious | Not serious | Not serious | Serious | 128 | 137 | -1.28  (-2.40; -0.16) | +???  VERY LOW | Important |
| *Sleep quality* | | | | | | | | | | | |
| 4 | RCT | Serious | Not serious | Not serious | Not serious | Serious | 116 | 100 | -1.35  (-1.79; -0.91) | ++??  LOW | Not important |
| *Stress* | | | | | | | | | | | |
| 3 | RCT | Serious | Not serious | Not serious | Not serious | Serious | 80 | 82 | -0.95  (-1.46; -0.43) | ++??  LOW | Not important |

Abbreviations (in alphabetical order): N: number; RCT: randomized controlled trial; SMD: standardized mean difference; WS: Withania somnifera; 95% CI: 95% confidence interval.

*Explanation of reasons for downgrading/upgrading:*

We GRADEd each pooled estimate for each relevant outcome according to the following criteria:

a. ***Risk of Bias:*** *We downgraded this domain by one level when any of the sources of Risk of Bias (as described above) were rated as “high”, or by half level when rated as “unclear”, for any of the studies included in the pooled estimate.*

b. ***Inconsistency:*** *We downgraded this domain by one level where the I^2^ value indicated substantial levels of heterogeneity (i.e., I^2^≥75%).*

c. ***Indirectness:*** *We protected against indirectness in this review by ensuring all studies included in any meta-analysis reported data from a validated psychometric tool. We therefore did not downgrade this domain for any assessment point.*

d. ***Imprecision:*** *We downgraded this domain by one level where the 95% confidence interval included the null value.*

e. ***Other Considerations:*** *We downgraded by one level when the number of studies contributing to the pooled estimate was <10 or in case of evidence of publication bias detected by the Egger’s test. However, if the effect estimate adjusted using the trim-and-fill method remained similar in magnitude and statistically significant, we did not downgrade, as this suggested that the results were robust to potential publication bias.*
